# Production and Quality Control of [^177^Lu]Lu-PSMA-I&T: Development of an Investigational Medicinal Product Dossier for Clinical Trials

**DOI:** 10.3390/molecules27134143

**Published:** 2022-06-28

**Authors:** Valentina Di Iorio, Stefano Boschi, Cristina Cuni, Manuela Monti, Stefano Severi, Giovanni Paganelli, Carla Masini

**Affiliations:** 1 IRCCS Istituto Romagnolo per lo Studio dei Tumori “Dino Amadori” IRST, 47014 Meldola, Italy; cristina.cuni@irst.emr.it (C.C.); manuela.monti@irst.emr.it (M.M.); stefano.severi@irst.emr.it (S.S.); giovanni.paganelli@irst.emr.it (G.P.); carla.masini@irst.emr.it (C.M.); 2 Department of Pharmacy and Biotechnologies, University of Bologna, 47921 Rimini, Italy; stefano.boschi@unibo.it

**Keywords:** prostate cancer, [^177^Lu]Lu-PSMA-I&T, IMPD, quality assurance

## Abstract

Since prostate cancer is the most commonly diagnosed malignancy in men, the theranostic approach has become very attractive since the discovery of urea-based PSMA inhibitors. Different molecules have been synthesized starting from the Glu-urea-Lys scaffold as the pharmacophore and then optimizing the linker and the chelate to improve functional characteristics. This article aimed to highlight the quality aspects, which could have an impact on clinical practice, describing the development of an Investigational Medicinal Product Dossier (IMPD) for clinical trials with [^177^Lu]Lu-PSMA-I&T in prostate cancer and other solid tumors expressing PSMA. The results highlighted some important quality issues of the final preparation: radiolabeling of PSMA-I&T with lutetium-177 needs a considerably longer time compared with the radiolabeling of the well-known [^177^Lu]Lu-PSMA-617. When the final product was formulated in saline, the stability of [^177^Lu]Lu-PSMA-I&T was reduced by radiolysis, showing a decrease in radiochemical purity (<95% in 24 h). Different formulations of the final product with increasing concentrations of ascorbic acid have been tested to counteract radiolysis and extend stability. A solution of 20 mg/mL of ascorbic acid in saline prevents radiolysis and ensures stability over 30 h.

## 1. Introduction

Prostate cancer (PCa) is the most commonly diagnosed malignancy in men worldwide and remains one of the leading causes of cancer-related deaths. Prostate-specific membrane antigen (PSMA) is a type II membrane glycoprotein with an extensive extracellular domain (44−750 amino acids) and plays a significant role in prostate carcinogenesis and progression [1,2,3,4].

PSMA expression correlates with the malignancy of the disease, being further increased in metastatic and hormone-refractory patients [5]. As a consequence, PSMA has attracted attention as a target for molecular imaging and targeted radioligand therapy, especially in metastatic castration-resistant prostate cancer (mCRPC).

Since the discovery of urea-based PSMA inhibitors in 2001 [6], a variety of PSMA-targeted radioligands for imaging prostate cancer was developed. Briefly, most of the relevant molecules are structured by three main components: a pharmacophore, usually X-urea-Glu (XuE)-scaffold, in our case X = K; a linker to enhance affinity by interaction with receptor hydrophobic pocket; and a chelator to bind the radionuclide. Other types of PSMA inhibitors are the ones labeled with fluorine-18 ([^18^F]F-PSMA-1007, ([^18^F]F-DCMPyL, and others), the chelator is in those cases replaced by organic structures with a leaving group to support an SN2 reaction. The “gold standard” ligand for PSMA imaging is Glu-NH-CO-NH-Lys(Ahx)-^68^Ga-HBED-CC ([^68^Ga]Ga-PSMA-HBED-CC) characterized by a high-affinity chelator for Gallium-68 (logK = 38.5) that seems to interact advantageously with the lipophilic part of the PSMA binding pocket [7]. Unfortunately, the HBED-CC chelator is unsuitable for radiolabeling with therapeutic radiometals such as yttrium-90 and lutetium-177, and therefore new theranostic compounds were designed to bind gallium-68, as well as yttrium-90 and lutetium-177.

Substitution of HBED-CC with DOTA and systematical modification of the side chain with the introduction of a naphtylic linker have led to PSMA-617 [8] with excellent pharmacokinetic properties, high binding affinity and internalization, prolonged tumor uptake, and high tumor-to-background ratio, which are extremely important for both imaging quality and therapy.

In parallel, another theranostic PSMA-targeted radioligand, PSMA-I&T, was explored [9]. The DOTAGA-FFK(SubKuE)-scaffold represents a flexible and adjustable backbone for the development of KuE-based PSMA inhibitors. Additionally, the DOTA chelator was substituted with DOTAGA (1,4,7,10-tetraazacyclododecane-1-(glutaric acid)-4,7,10-triacetic acid) to facilitate the yttrium-90 and lutetium-177-labeling procedure, improve pharmacokinetics and, potentially, affinity to the receptor. DOTAGA derivatives showed higher hydrophilicity (logP = −3.9 ± 0.1 for DOTAGA 177Lu derivative compared with −2.7 ± 0.02 for that DOTA derivative) and improved affinity to PSMA, compared with DOTA-coupled counterpart, resulting in about a twofold-increased specific internalization of the ^68^Ga- and ^177^Lu-labelled DOTAGA analogue [10]. The substitution of one of the D-phenylalanine residues in the peptidic linker by 3-iodo-D-tyrosine improved the interaction of the tracer molecule with a remote binding site. These modifications, together with increasing the lipophilic interaction of the tracer with the PSMA enzymes, led to the second-generation theranostic tracers DOTAGA-(I-y), fk(Sub-KuE), and the PSMA I&T [9].

In order to use a radiopharmaceutical in human applications, it should be manufactured under Good Manufacturing Practice (GMP) or National Regulations. In Italy, the reference quality assurance system is “Norme di Buona Preparazione dei Radiofarmaci per Medicina Nucleare (NBP-MN)” [11], which is a GMP-like quality assurance system dealing with no-profit clinical trials.

To submit the clinical study to the Italian Medicines Agency (AIFA), an Investigational Medicinal Product Dossier (IMPD) needs to be produced for [^177^Lu]Lu-PSMA-I&T according to European Medicines Agency EMA guideline [12]. This guideline aims to address the documentation on the chemical and pharmaceutical quality of investigational medicinal products (IMPs), including radiopharmaceuticals, to ensure their quality, safety, and efficacy.

The aim of this paper is to describe the process validation as well as the analytical methods, along with establishing acceptance criteria for [^177^Lu]Lu-PSMA-I&T according to the purpose of obtaining an IMPD.

At the moment the ligand PSMA-I&T is commercially available from ABX and there are no patents that prevent us from working with PSMA-I&T.

## 2. Results

IMPD for [^177^Lu]Lu-PSMA-I&T was prepared according to the EMA guideline [12]. IMPD includes the most up-to-date information relevant to the clinical trial available at the time of submission of the clinical trial application. It essentially consists of two parts, the first dedicated to the drug substance and the second dedicated to the investigational medicinal product under test.

### 2.1. Drug Substance

Two drug substances have been identified: the ligand PSMA-I&T and the precursor [^177^Lu]LuCl_3_.

#### 2.1.1. PSMA-I&T

Nomenclature:

(R)-DOTAGA-D-Tyr(3-I)-D-Phe-D-Lys[Sub-Lys-CO-Glu]-OH (supplied as acetate salt)
Synonyms (R)-DOTAGA-(I-y)fk(Sub-KuE) Structure
Molecular formula: C_63_H_92_IN_11_O_23_
Molecular weight: 1498.37 g/mol

**Figure 1:**
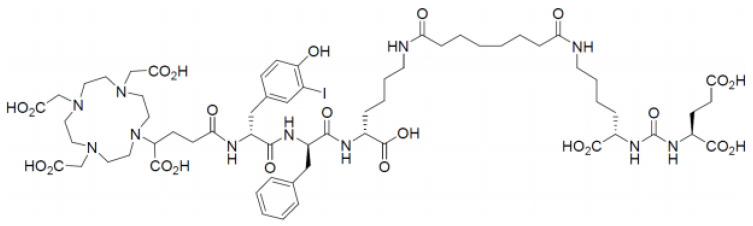
The chemical structure of PSMA- I&T.

The chemical structure of PSMA-I&T is showed in Figure 1.

#### 2.1.2. Lutetium-177

PSMA-I&T was radiolabeled with lutetium-177 chloride. Lutetium-177 chloride was produced under a marketing authorization (MA) by irradiation of highly enriched (>99%) ytterbium-176 by a neutron source.

Lutetium-177 was produced according to the following nuclear reaction:

[^176^Yb(n,γ)^177^Yb → (β^-^) → ^177^Lu]

The neutron thermal flux was between 1013 and 1016 cm^−2^ s^−1^. The nuclear reaction is no-carried added (n.c.a.). The n.c.a. reaction results in a very high specific activity (≥3000 GBq/mg), in comparison with the lower specific activity (500 GBq/mg) when the Lutetium-177 is obtained by neutron irradiation of Lutetium-176. Moreover, the “direct production” leads to formation of a radionuclidic impurity (Lutetium-177m), not present in the Lutetium-177 produced by ytterbium-176. Metallic impurities concentration was very low, the sum of both metallic as well as other radionuclidic impurities cannot interfere with labeling nor with radiochemical and radionuclidic purity.

The specifications for the release of lutetium-177 chloride are indicated in Table 1.

### 2.2. Investigational Medicinal Product (IMP) under Test

#### Description and Composition of the IMP

The IMP consists of a description of the [^177^Lu]Lu-PSMA-I&T solution, among other things stating the range of radioactivity (17,980–29,790 MBq), at the end of synthesis (EOS), which, in this case, is also considered Activity Reference Time (ART); the final volume is 17–25 mL.

The radioactive concentration is between 1057 and 1192 MBq/mL. IMP is formulated as a multidose drug with the components described in Table 2.

**Figure 2:**
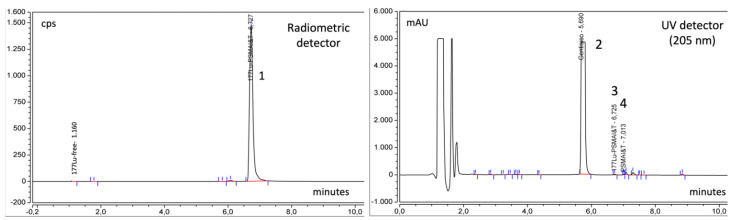
Relevant chromatograms of the final products of [^177^Lu]Lu-PSMA-I&T with radiometric and UV detector: Peak 1 = [^177^Lu]Lu-PSMA-I&T, Peak 2 = Gentisic acid, Peak 3 = [^177^Lu]Lu-PSMA-I&T, Peak 4 = PSMA-I&T.

Typical radiometric and UV chromatograms of [^177^Lu]Lu-PSMA-I&T syntheses are shown in Figure 2, which shows the difference between the retention time of [^177^Lu]Lu-PSMA-I&T and the precursor PSMA-I&T (6.72 and 7.01, respectively). The largest peak in the UV chromatogram is gentisic acid. The radiometric detector evidences the high radiochemical purity of the product, since only the peak of [^177^Lu]Lu-PSMA-I&T is present.

### 2.3. Quality Controls

#### 2.3.1. Acceptance Criteria

Acceptance criteria, specifications, and release timing were chosen in compliance with the general texts and monographs of the current European Pharmacopoeia and are summarized in Table 3. The product should meet the acceptance criteria for all the established quality parameters. The administered patient dose ranged from 5500 to 7400 MBq according to the patient conditions.

All the tests, except sterility, were carried out before the release.

#### 2.3.2. Validation of the Analytical Procedures

Validation is the act of proving that any procedure, process, equipment, material, activity, or system actually leads to the expected results, with the aim to contribute to and guarantee the quality of a radiopharmaceutical.

The objective of validation of an analytical procedure is to demonstrate that it is suitable for its intended purpose.

The validation of the analytical procedures, the acceptance limits, and the parameters (specificity, linearity, range, accuracy, precision, quantification, and detection limit) for performing validation of analytical methods was carried out according to the ICH guideline Q2(R1) [13]. For the HPLC determination of chemical purity, ^nat^Lu-PSMA-I&T and PSMA-I&T were used. The high concentration and absorbance of gentisic acid cause it to be by far the highest peak in the UV (205 nm) spectrum of the PSMA-I&T product; we, therefore, chose to perform the validation of the PSMA-I&T analytical method in presence of gentisic acid to simulate closely the condition of the final product formulation. Parameters and acceptance criteria for the validation of the radio-HPLC method and the results obtained are shown in Table 4. Radionuclidic purity, [^177^Lu]Lu-PSMA-I&T is verified on the basis of the certificate of analysis attached by the supplier of the [^177^Lu]LuCl_3_.

**Figure 3:**
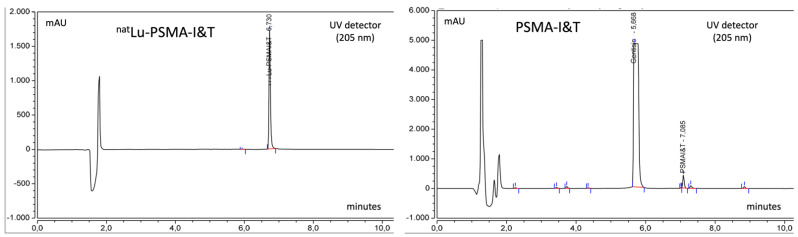
Chromatograms of standard solutions of ^nat^Lu-PSMA-I&T (20 μL of a 0.1 mg/mL solution) and PSMA-I&T (20 μL of a 0.04 mg/mL solution). Rt of ^nat^Lu-PSMA-I&T is slightly lower than that Rt of [^177^Lu]Lu-PSMA-I&T with radiometric detection because it is positioned after the UV detector.

Chromatograms of standards ^nat^Lu-PSMA-I&T and PSMA-I&T are shown in Figure 3.

#### 2.3.3. Bioburden

The pre-filtrated product was sent for a bioburden test, using 1 mL for each test sample. (Eurofins Laboratory Biolab Srl, Vimodrone, Milan Italy.)

The results were:

Total aerobic microbial count (TAMC) < 1 cfu/mL,
Total yeast and mold count (TYMC) < 1 cfu/mL,
where <1 cfu/mL means absence of colonies.

#### 2.3.4. Batch Analysis and Process Validation

Process Validation should be intended as a means to establish that all the process parameters that bring to the preparation of the intended radiopharmaceutical and their quality characteristics are consistently and reproducibly met.

Process validation was carried out by producing three different batches of [^177^Lu]Lu-PSMA-I&T on three different days, in the same conditions set for typical routine preparations and in the activity range reported in the acceptance criteria. Each batch was prepared accordingly the validation protocol should be fully characterized from the analytical point of view, with the aim to verify that the product meets the acceptance criteria as for all the established quality parameters.

Parameters were measured by the tests described in Table 3.

The results for three representative batches are shown in Table 5. 

All the batches used for process validation complied with the acceptance criteria.

#### 2.3.5. Stability

Stability was assessed at 0, 24, and 30 h after the end of the synthesis. The three batches used for process validation were kept at room temperature and then parameters that could change over time such as appearance, radiochemical purity, and pH were reanalyzed after 24 and 30 h. The synthesis does not affect the radionuclidic purity so the radionuclidic purity is the same as observed on the sheet of [^177^Lu]LuCl_3_.

The analysis of the radiochemical purity was carried out by HPLC and TLC because the TLC method is used to detect the presence of colloidal lutetium-177 [14] that is not detectable with HPLC analysis.

**Figure 4:**
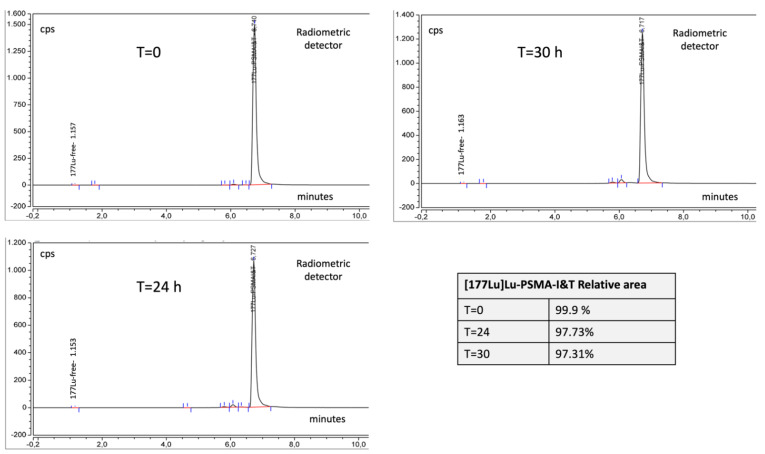
[^177^Lu]Lu-PSMA-I&T (radiometric detector) during stability studies, at T = 0, T = 24 h, T = 30. In the table inside the figure are reported relative areas of [^177^Lu]Lu-PSMA-I&T as a percentage of the total areas.

For organizational reasons, it is not possible to administer the drug to the patient, later than 24 h after preparation. Chromatograms are shown in Figure 4; the stability data are shown in Table 6. 

## 3. Discussion

[^68^Ga]Ga-PSMA-HBED-CC [7] represents a breakthrough in the imaging and staging of PCa. To meet the clinical need for a therapeutic agent for treatment of PCa, some promising urea-based candidates have been investigated, [^177^Lu]Lu-PSMA-617 and [^177^Lu]Lu-PSMA-I&T [8,9]. The two molecules have the same pharmacophore but different linkers and chelators.

The effect of replacing the DOTA chelator with DOTAGA led to an increase in radiolabeling reaction time: PSMA-I&T was incubated with [^177^Lu]LuCl_3_ at 95 °C for 30 min. to obtain [^177^Lu]Lu-PSMA-I&T instead of 8 min at 100 °C for [^177^Lu]Lu-PSMA-617. The longer reaction time for the incorporation of Lutetium-177 in the DOTAGA is in agreement with Weineisen et al. [9] and could probably be ascribed to a slower kinetic of incorporation due to different conformational changes in the chelators along with the overall molecular structure of the linker.

The two molecules showed slight differences in solubility characteristics, which, however, did not require substantial changes in the buffer solutions.

The longer incubation time needed to prepare [^177^Lu]Lu-PSMA-I&T did not affect the impurities profile. In terms of quality parameters, the experimental results from the three batches of [^177^Lu]Lu-PSMA-I&T fulfilled the specifications. Furthermore, the radiolabeling conditions always led to a very high radiochemical yield.

To prevent radiolysis of the radiopharmaceutical, the radiolabeling was carried out in presence of ascorbic and gentisic acid.

The tests performed for quality controls are commonly used in radiochemical and radiopharmaceutical methods.

HPLC allows the use of two detectors in series for example UV or a mass detector coupled with a radiometric detector.

Acceptance criteria are based on the Eu. Pharm. general monograph “Radiopharmaceutical Preparation” [15] and on National Regulations for preparation of radiopharmaceuticals [11].

Gamma-ray spectroscopy is used for the measurement of the radionuclidic purity of radiopharmaceuticals. The Pharmacopoeia generally states that radiopharmaceuticals should have a radionuclidic purity of at least 99.9% throughout their shelf-life. High purity germanium detectors are required to detect impurities of less than 0.1%. For identification, the same approach was used to compare γ energies lines characteristics of the radionuclide.

The most significant quality issue in the preparation of [^177^Lu]Lu-PSMA-I&T is the poor stability of the finished product when diluted with saline to a final volume of 17–25 mL, a general practice with other radiopharmaceuticals such as [^177^Lu]Lu-PSMA-617. Comparative results on the stability of both radiopharmaceuticals are reported in Table 7.

[^177^Lu]Lu-PSMA-I&T was found to be instable in saline solution (<95% radiochemical purity after 24 h). These data emphasize that what is reported in the Supplemental data by Weineisen et al. [9] either does not consider the long-term stability of the preparation or establishes less stringent acceptance criteria compared with this paper (≥97% radiochemical purity). Another aspect to consider is the large difference between the final radioactivities reported in the literature [9] and the radioactivities used in this study. Long-term radiolysis due to high radioactivity concentration should be taken into account. [^177^Lu]Lu-PSMA-617, in our experience, is stable in saline; this behavior can probably be ascribed to [^177^Lu]Lu-PSMA-I&T and [^177^Lu]Lu-PSMA-617 having different linkers that are affected differently by radiolysis.

To overcome this problem, [^177^Lu]Lu-PSMA-I&T was formulated in an ascorbic 20mg/mL saline solution to a final volume of 17–25 mL, resulting in a shelf life of 30 h, which is the optimal time interval for the management of patients.

## 4. Materials and Methods

### 4.1. [^177^Lu]Lu-PSMA-I&T Manufacturing Process and Process Controls

For the radiolabeling of [^177^Lu]Lu-PSMA-I&T, a manual synthesis was used. The radiosynthesis was carried out in a shielded isolator offering a class A environment with class B pre-chambers (Manuela Beta, COMECER S.p.A, Castelbolognese, Italy) located in a class C cleanroom.

#### 4.1.1. Reagents

[^177^Lu]LuCl_3-_EndolucinBeta, radiopharmaceutical precursor with a MA was obtained from ITM Medical Isotope GmbH, Germany—and was supplied by Gamma Servizi S.r.l, Borgarello, Italy

PSMA-I&T GMP precursor vials 1 mg purchased from ABX Advanced Biochemical Compounds Biomedizinische Forschungsreagenzien GmbH Radeberg, Germany.

Water for Injectable Preparations (100 mL bottles) with a MA was purchased from Monico S.p.A. Venezia/Mestre, Italy.

Gentisic acid, (97.5–102.5% purity), was supplied by Merck KGaA, Darmstadt, Germany.

Ascorbic acid, (99.0–100.5% purity), was supplied by VWR International, Leuven, Belgium.

NaOH, (≥99% purity), was supplied by Merck KgaA, Darmstadt, Germany.

Anhydrous sodium acetate, (≥99% purity), was supplied by Merck KgaA, Darmstadt, Germany.

Gentisic acid/ascorbic acid buffer pH = 5 was prepared by IRST Radiopharmacy prior to the radiolabeling. The buffer was prepared with 3.1 g of sodium acetate, 1.6 g of gentisic acid, and 3.0 g of ascorbic acid, which were dissolved in 46 mL of water for injectable preparations measured with a 60 mL sterile syringe. This solution was pH adjusted by the addition of 11.5 mL of a 2N NaOH solution. The final pH was 5.2 ± 0.1 and was verified by pH meter. This solution was sterile filtered through a single-use, sterile, pyrogen-free 0.22 μm ventilated filter (Vented Millex-GV SLGV255F Merck Millipore Ltd.). The buffer was aliquoted into ten 1 mL fractions, which were stored at −20 °C. Shelf life of the laboratory-prepared labeling buffer solution was 1 month.

Ascorbic acid-Vitamin C SALF 1000 mg/5 mL solution for injection vials with an MA was purchased from S.A.L.F Laboratorio Farmacologico, Cenate Sotto Bergamo, Italy.

Sodium Chloride 0.9% 100 mL, with a MA was purchased from Fresenius Kabi S.r.l., Isola della Scala, Italy.

Sterile glass vials under partial vacuum—manufactured by Eckert&Ziegler GmbH, Berlin, Germany—were purchased by Radius S.r.l. Budrio, Italy.

#### 4.1.2. Manufacturing of [^177^Lu]Lu-PSMA-I&T

**Figure 5:**
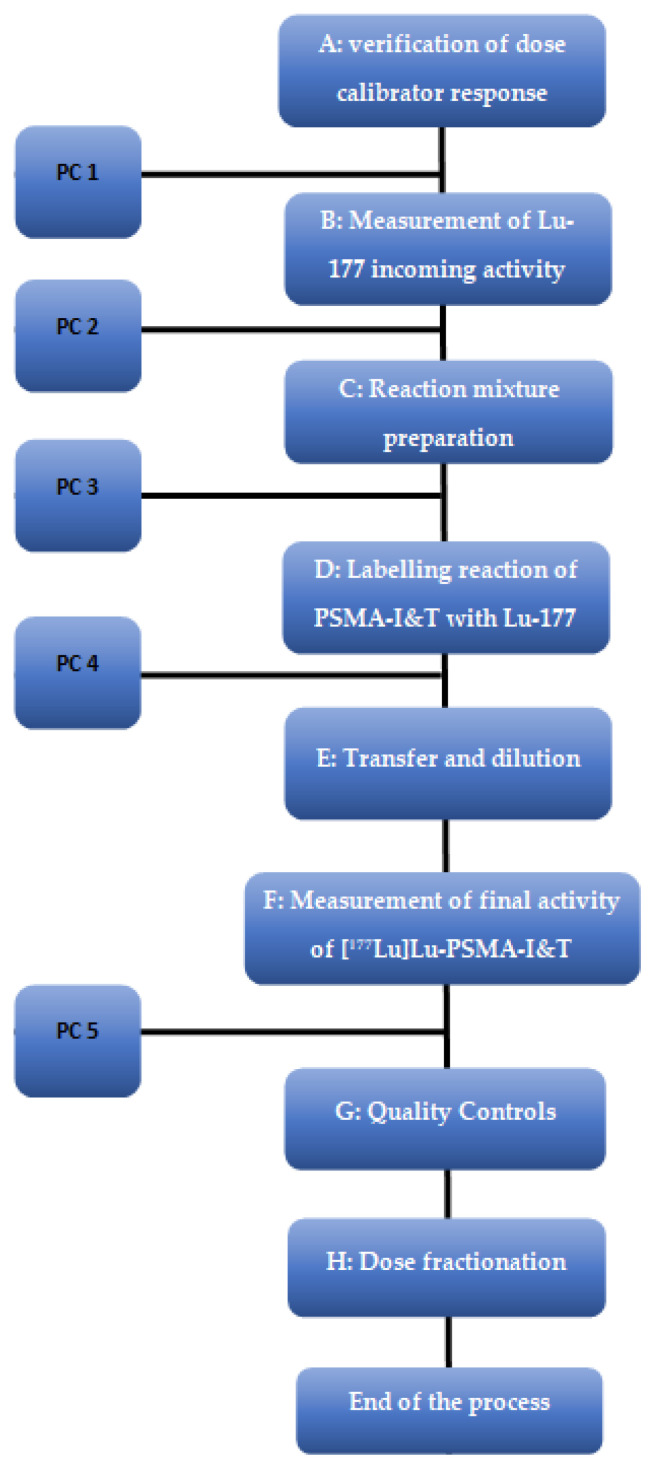
Flow chart of the radiolabeling of PSMA-I&T. PC = In-Process Control.

The precursor was incubated with lutetium-177 at 95 °C for 30 min in the presence of ascorbic and gentisic acid. A typical synthesis time for the complexation reaction yielding [^177^Lu]Lu-PSMA I&T in 60 min with a radiochemical yield of 96.8 ± 0.9% (*n* = 5). The flow chart of the radiolabeling of PSMA-I&T is shown in Figure 5.

Step A—Verification of dose calibrator response using a certified source of cesium-37 (NuklearMedizin, Dresden, Germany). The deviation between the measured and expected value should never be greater than 5%.

Step B—The radioactivity of the received [^177^Lu]LuCl_3_ vial using dose calibrator.

Step C—The reaction mixture was prepared with 0.5–0.8 mL of PSMA-I&T (in water) and 0.5 mL of gentisic–ascorbic acid solution. The amount of PSMA-I&T should be adequate to obtain the specific activity (SA) of 36–39 GBq/mg. The reaction mixture is set up in a 2.5 mL syringe in the shielded isolator immediately before radiolabeling.

Step D—PSMA-I&T solution was added to the [^177^Lu]LuCl_3_ vial (0.4–0.8 mL). The vial was placed in the heater, at 95 °C with continuous temperature monitoring for 30 min, to help the complexation reaction to take place.

Step E—The vial containing a volume of about 1.5 mL of [^177^Lu]Lu-PSMA-I&T was diluted by adding 15–23 mL of an ascorbic acid solution (20 mg/mL) to the reaction vial. The reactor vial is connected to the product vial via a sterile needle, tubing, and a 0.22 µm ventilated sterile filter. The needle in the reaction vial needs to be at the bottom of the reaction vial. The pressure created in the reaction vial pushed the solution ca. 16–25 mL into the product vial.

#### 4.1.3. In Process Controls (PC)

PC 1: Accuracy testing of emitting β-sources is performed prior to the introduction of a new measured geometry (e.g., new vial size of lutetium-177).

PC 2: A daily radioactivity check is performed before each production run and the deviation between the read and calculated activity, according to the calibration certificate, must be <±10%.

PC 3: Operator aseptic work techniques are verified by media fill test.

PC 4: Double check of the temperature.

PC 5: Control of the final product activity; the radiochemical yield is also calculated.

### 4.2. Quality Control

#### 4.2.1. Standard Procedures

pH was determined by pH strips (Merck pH indicator strip, Acilit, increment 0.5 pH unit).

The Endotoxin test was performed by the Limulus amebocyte lysate test (LAL test) on an Endosafe Nexgen-PTS™ (Charles River Laboratories Italia, Calco, Italy).

Since this is a preparation that cannot be subjected to terminal sterilization, the product solution, therefore, has to be subjected to sterile filtration through a sterile filter (pores size less than 0.22 µm).

Filter integrity must be checked by bubble point test before the release. The bubble point test was performed on an Integritest 4 system (Merck Millipore, Merck KgaA, Darmstadt, Germany.

A 1 mL aliquot of the product mixture was sent to the Microbiological Laboratory of the Regional Healthcare, Pievesestina, Cesena, Italy for the sterility tests. The sterility test was performed according to current European Pharmacopoeia Monograph 2.6.1 “Sterility”.

#### 4.2.2. HPLC Analysis

^nat^Lu-PSMA-I&T was purchased from ABX GmbH—Advanced Biochemical Compounds (Radeberg, Germany) as a reference standard.

HPLC analysis was performed on an Ultimate 3000 system equipped by a UV variable wavelength detector RS300 (Thermo Fischer Scientific, Germany) and a radiometric detector (GABI, Raytest, Germany). The system was run by Chromeleon software version 7.2 SR5 (Dionex Sunnyvale, CA, USA).

The column was an Acclaim 120 C18, 3 µm, 120Å, 3 × 150 mm (Thermo Scientific, Waltham, MA, USA).

A multi-step gradient was applied using two solvent A (0.1% TFA in water) and solvent B (0.1% TFA in acetonitrile): 92% A to 40% A in 10 min, then from 40% A to 20% A for a further 3 min, and back to 92% A in 2 min, then stable for 5 min. The flow rate was set at 0.6 mL/min. UV wavelength at 205 nm. Column Oven: 25 °C. Injection volume 20 µL. Retention times of ^nat^-LuPSMA-I&T and PSMA-I&T were 6.730 and 7.013, respectively.

Chemical purity was calculated by comparing the areas of the peaks of the product with a standard solution injected before the analysis of the final product, as a general practice suggested by Pharmacopoeia.

#### 4.2.3. Thin-Layer Chromatography (TLC)

TLC was performed using a TLC Silica Gel 60 (Merck KGaA, Darmstadt, Germany), Approximately 1–2 μL of IMP injection solution was spotted on the plate. The solvent for the development of the TLC plates was ammonium acetate 0.1N and methanol 50:50 *v*/*v*. The developed plate was analyzed by autoradiography on MS (MultiSensitive) storage phosphor screens and by a Cyclone Plus Storage Phosphor System (PerkinElmer).

Rf of lutetium-177 free and colloids was 0.2; Rf of [^177^Lu]Lu-PSMA-I&T was 0.8.

## 5. Conclusions

This study demonstrates that [^177^Lu]Lu-PSMA-I&T can be prepared as a radiopharmaceutical suitable for human use. Clearly defined acceptance criteria, validations plans, and methods for quality control were outlined. We compared two structural related radiopharmaceuticals, which differed in chelator and linker, but not in pharmacophore. The difference resulted in a different metal chelation kinetic, a different solubility, and, above all, a very different stability of the two radiopharmaceuticals. Ascorbic acid functions as a scavenger and can significantly prolong the shelf life of [^177^Lu]Lu-PSMA-I&T by at least 30 h, and can prolong the time interval where patients can be treated.

## 6. Patents

PSMA-I&T is available by ABX and there are no patents.

## Figures and Tables

**Table 1:** Specifications of lutetium-177 chloride.

Test	Method	Specification	Unit
[^177^Lu]LuCl_3_ in HCl 0.04M pH 1–2 Activity per Vial value decay corrected to ART	n.a.	90–110 of the activity stated in the label	%
Volume delivered	n.a.	0.4–0.8 mL According to the radioactivity ordered	mL
Appearance	Visual test	Clear and colorless solution	n.a.
Identity Lu-177	Gamma Spectrometry	113 KeV gamma line 208 KeV gamma line	n.a.
Identity Chloride	Eu. Phar.	White precipitate visible	n.a.
Specific activity value decay corrected to ART	ICP-MS	≥3000	GBq/mg
Radionuclidic purity Radiochemical purity	Gamma Spectrometry TLC	Yb-175 ≤ 0.01 Sum of impurities ≤ 0.01 ≥99.0 as ^177^LuCl_3_	% % %
Chemical purity	ICP-MS	Fe ≤ 0.25 Cu ≤ 0.5 Zn ≤ 0.5 Pb ≤ 0.5 Yb-176 ≤ 0.1 Sum of impurities ≤ 0.5	µg/GBq
Radiolabeling yield	TLC	≥99.0	%
Sterility	Eu. Phar.	Sterile	n.a.
Bacterial endotoxins	Eu. Phar.	≤20	EU/mL

ART = Activity reference time, ICP-MS = Inductively coupled plasma–mass spectrometry, TLC = Thin layer chromatography, Eu.Phar. = European Pharmacopoeia. Radiolabeling yield (TLC): based on radiolabeling Lu-177 of DOTA-derivated molar ratio 1:4 (CoA).

**Table 2:** Batch formula of [^177^Lu]Lu-PSMA-I&T.

Components	Function	Amount/Activity
[^177^Lu]LuCl_3_	Active Pharmaceutical Ingredient (API)	18,350–31,040 MBq Activity Reference Time (ART)
PSMA-I&T	Precursor	500–800 µg (334–534 nmol)
Water for injection	For reconstitution of PSMA-I&T	0.5–1 mL
Gentisic/ascorbic buffer composition:		
Gentisic acid	Radical scavenger	16.8 mg (109 µmol)
Sodium acetate	Buffer solution	32.4 mg (395 µmol)
Sodium hydroxide	pH balance buffer	9.6 mg (240 µmol)
Ascorbic acid	Radical scavenger	31.2 mg (177 µmol)
Ascorbic acid solution in NaCl 0.9% 20 mg/mL	Diluent and radical scavenger	17–25 ml

**Table 3:** Recommended test for the quality controls.

Parameter	Method	Acceptance Criteria
[^177^Lu]Lu-PSMA-I&T activity	Dose calibrator	17,980–29,790 MBq
Radioactive concentration	Dose calibrator	1057–1192 MBq/mL
Volume		17–25 mL
Appearance	Visual test	Clear and colorless solution
Identification	HPLC	_Rt_ [^177^Lu]Lu-PSMA-I&T ± 0.2 min vs. Rt ^nat^Lu-PSMA-I&T reference standard
Radionuclidic identity	Gamma Spectrometry	113 KeV gamma line 208 KeV gamma line
Yb-175 content	Gamma Spectrometry	Yb-175 ≤ 0.01%
Radiochemical purity	HPLC	[^177^Lu]Lu ≤ 3% [^177^Lu]Lu-PSMA-I&T ≥ 97%
Radiochemical purity	TLC	[^177^Lu]Lu colloids ≤ 3% [^177^Lu]Lu-PSMA-I&T ≥ 97%
pH	pH strips	4.5–5.5
Filter integrity	Bubble Point Test	≥50 psi
Sterility	Sterility Test (Eur. Ph.)	Sterile
Bacterial endotoxins	Eur. Ph.	≤175 EU/V

PSMA-I&T ≤ 0.1 mg/V_max_ where Vmax is the maximum injectable volume of the preparation. Volume is determined by the sum of the volume of the reagents and the volume of Ascorbic acid solution in NaCl 0.9% 20 mg/mL added to dilute the final product.

**Table 4:** Parameters and acceptance criteria for the validation of the radio-HPLC method and the obtained results.

Chemical Purity UV Detector		
Parameters	Acceptance Criteria	Results
Specificity	Rs ^nat^LuPSMA I&T and PSMA-I&T Rs ≥ 1.5	Comply
Precision	CV% PSMA-I&T ≤ 5% CV% ^nat^LuPSMA-I&T ≤ 5%	<4% <3%
Linearity	R^2^ PSMA-I&T ≥ 0.99 R^2 nat^LuPSMA-I&T ≥ 0.99	≥0.999 >0.999
LOQ (µg/mL)	Experimental	PSMA-I&T = 6.8 ^nat^LuPSMA-I&T = 13.2
LOD (µg/mL)	Experimental	PSMA-I&T = 2.2 ^nat^LuPSMA-I&T = 4.3
Range	80–120%	Comply
Accuracy	Average bias < 5%	Comply
**Radiochemical Purity Radiodetector**		
Parameters	Acceptance Criteria	Results
Specificity	Difference t_R_ ± 5% RT compared with RT ^nat^LuPSMA-I&T	±4%
Precision	CV% ≤ 5%	≤3.2%
Linearity	R^2^ ≥ 0.99	≥0.999
LOQ	n.a.	n.a.
LOD	n.a.	n.a.
Range	n.a.	n.a.

Rs = Resolution, CV= coefficient of variation, R^2^ = correlation coefficient, LOQ = quantitation limit, LOD = detection limit, n.a. = not applicable.

**Table 5:** Results of [^177^Lu]Lu-PSMA-I&T representative batches.

Parameter	Method	Acceptance Criteria	Batch 08/04/2021	Batch 15/04/2021	Batch 07/05/2021
[^177^Lu]Lu-PSMA-I&T Activity	Dose Calibrator	17,980–29,790 MBq	17,980 MBq	21,720 MBq	29,790 MBq
Radioactive concentration	Dose Calibrator	1057–1192 MBq/mL	1058 MBq/mL	1086 MBq/mL	1192MBq/mL
Volume	-	17–25 ml	17 mL	20 mL	25 mL
Appearance	Visual test	Clear and Colorless Solution	Complies	Complies	Complies
Identification	HPLC	Rt [^177^Lu]Lu-PSMA-I&T ± 0.2 min vs. Rt ^nat^Lu-PSMA-I&T reference standard	+0.012	+0.03	+0.01
Radionuclidic identity	Gamma Spectrometry	113 KeV gamma line 208 KeV gamma line	Comply	Comply	Comply
Yb-175 content	Gamma Spectrometry	Yb-175 ≤ 0.01%	Comply	Comply	Comply
Radiochemical purity	TLC	[^177^Lu]Lu colloids ≤ 3% [^177^Lu]Lu-PSMA-I&T ≥ 97%	0 100%	0 100%	0 100%
Radiochemical purity	HPLC	[^177^Lu]Lu ≤ 3% [^177^Lu]Lu-PSMA-I&T ≥ 97%	0.02% 99.3%	0.02% 99.4%	0.01% 99.4%
Chemical purity	HPLC	PSMA-I&T ≤ 0.1 mg/Vmax Sum of impurities ≤ 0.5 mg/Vmax	Complies	Complies	Complies
pH	pH Strips	4.5–5.5	5	5	5
Filter integrity	Bubble Point Test	≥50 psi	≥50 psi	≥50 psi	≥50 psi
Sterility	Sterility Test (Eur. Ph.)	Sterile	Sterile	Sterile	Sterile
Bacterial endotoxins	Eur. Ph.	≤175 EU/V	≤10 EU/V	≤10 EU/V	≤10 EU/V

Rt = retention time.

**Table 6:** Stability data of [^177^Lu]Lu-PSMA-I&T at T0 and after 24 and 30 h at room temperature.

T0 Stability Test					
Parameter	Method	Acceptance Criteria	Batch 08/04/2021	Batch 15/04/2021	Batch 07/05/2021
Appearance	Visual test	Clear and Colorless Solution	Complies	Complies	Complies
Radiochemical purity	TLC	[^177^Lu]Lu colloids ≤ 3% [^177^Lu]Lu-PSMA-I&T ≥ 97%	0 100%	0 100%	0 100%
Radiochemical purity	HPLC	[^177^Lu]Lu ≤ 3% [^177^Lu]Lu-PSMA-I&T ≥ 97%	0.02% 99.3%	0.02% 99.4%	0.01% 99.4%
pH	pH Strips	4.5–5.5	5	5	5
**24 h stability test**					
**Parameter**	**Method**	**Acceptance** **Criteria**	**Batch** **08/04/2021**	**Batch** **15/04/2021**	**Batch** **07/05/2021**
Appearance	Visual test	Clear and Colorless Solution	Complies	Complies	Complies
Radiochemical purity	TLC	[^177^Lu]Lu colloids ≤ 3% [^177^Lu]Lu-PSMA-I&T ≥ 97%	0.1% 99.9%	0.1% 99.9%	0.1% 99.9%
Radiochemical purity	HPLC	[^177^Lu]Lu ≤ 3% [^177^Lu]Lu-PSMA-I&T ≥ 97%	0.01% 98%	0.02% 98%	0.04% 97.7%
pH	pH Strips	4.5–5.5	5	5	5
**30 h stability test**					
**Parameter**	**Method**	**Acceptance** **Criteria**	**Batch** **08/04/2021**	**Batch** **15/04/2021**	**Batch** **07/05/2021**
Appearance	Visual Test	Clear and Colorless Solution	Complies	Complies	Complies
Radiochemical purity	TLC	[^177^Lu]Lu colloids ≤ 3% [^177^Lu]Lu-PSMA-I&T ≥ 97%	0.1% 99.9%	0.2% 99.8%	0.1% 99.9%
Radiochemical purity	HPLC	[^177^Lu]Lu ≤ 3% [^177^Lu]Lu-PSMA-I&T ≥ 97%	0.04% 97.5%	0.04% 97.4%	0.07% 97.3%
pH	pH Strips	4.5–5.5	5	5	5

**Table 7:** Comparative stability data of [^177^Lu]Lu-PSMA-I&T and [^177^Lu]Lu-PSMA-617, diluted with saline, without ascorbic acid, at T = 0 and after 24 and 30 h at room temperature.

		T0 Stability Test		
Parameter	Method	Acceptance Criteria	[^177^Lu]Lu-PSMA-I&T	[^177^Lu]Lu-PSMA-617
Radiochemical purity	TLC	[^177^Lu]Lu colloids ≤ 3% [^177^Lu]Lu-PSMA-I&T ≥ 97%	0.3% 99.7%	0 100%
Radiochemical purity	HPLC	[^177^Lu]Lu ≤ 3% [^177^Lu]Lu-PSMA-I&T ≥ 97%	0.2% 99.1%	0.02% 99.8%
		**24 h stability test**		
**Parameter**	**Method**	**Acceptance** **Criteria**	**[^177^Lu]Lu-PSMA-I&T**	**[^177^Lu]Lu-PSMA-617**
Radiochemical purity	TLC	[^177^Lu]Lu colloids ≤ 3% [^177^Lu]Lu-PSMA-I&T ≥ 97%	3.3% 96.7%	0.1% 99.9%
Radiochemical purity	HPLC	[^177^Lu]Lu ≤ 3% [^177^Lu]Lu-PSMA-I&T ≥ 97%	0.2% 94.6%	0.04% 97.4%
		**30 h stability test**		
**Parameter**	**Method**	**Acceptance** **Criteria**	**[^177^Lu]Lu-PSMA-I&T**	**[^177^Lu]Lu-PSMA-617**
Radiochemical purity	TLC	[^177^Lu]Lu colloids ≤ 3% [^177^Lu]Lu-PSMA-I&T ≥ 97%	3.5% 96.5%	0.2% 99.8%
Radiochemical purity	HPLC	[^177^Lu]Lu ≤ 3% [^177^Lu]Lu-PSMA-I&T ≥ 97%	0.2% 93.2%	0.06% 97.2%

## Data Availability

The data presented in this study are available on request from the corresponding author.

## Sample Availability

Samples of the compounds are not available from the authors.
